# Regulation and Function of Chemokines at the Maternal–Fetal Interface

**DOI:** 10.3389/fcell.2022.826053

**Published:** 2022-07-22

**Authors:** Sainan Zhang, Jinli Ding, Yan Zhang, Su Liu, Jing Yang, Tailang Yin

**Affiliations:** ^1^ Reproductive Medical Center, Renmin Hospital of Wuhan University & Hubei Clinic Research Center for Assisted Reproductive Technology and Embryonic Development, Wuhan, China; ^2^ Department of Clinical Laboratory, Renmin Hospital of Wuhan University, Wuhan, China; ^3^ Shenzhen Key Laboratory of Reproductive Immunology for Peri-implantation, Shenzhen Zhongshan Institute for Reproduction and Genetics, Shenzhen Zhongshan Urology Hospital, Shenzhen, China

**Keywords:** chemokine, chemokine receptor, trophoblast, decidual cells, maternal–fetal interface, pregnancy

## Abstract

Successful pregnancy requires the maternal immune system to tolerate the semi-allogeneic embryo. A good trophoblast function is also essential for successful embryo implantation and subsequent placental development. Chemokines are initially described in recruiting leukocytes. There are rich chemokines and chemokine receptor system at the maternal–fetal interface. Numerous studies have reported that they not only regulate trophoblast biological behaviors but also participate in the decidual immune response. At the same time, the chemokine system builds an important communication network between fetally derived trophoblast cells and maternally derived decidual cells. However, abnormal functions of chemokines or chemokine receptors are involved in a series of pregnancy complications. As growing evidence points to the roles of chemokines in pregnancy, there is a great need to summarize the available data on this topic. This review aimed to describe the recent research progress on the regulation and function of the main chemokines in pregnancy at the maternal–fetal interface. In addition, we also discussed the potential relationship between chemokines and pregnancy complications.

## 1 Introduction

Pregnancy is a complex and highly coordinated physiological process. Successful pregnancy involves the cooperation of multi-step crucial events at the maternal–fetal interface. First, at the early stages of pregnancy, the proper invasion, proliferation, and differentiation function of trophoblast cells are particularly important for achieving placental formation and embryonic development (Burrows et al., 1996; Anin et al., 2004; Staun-Ram and Shalev, 2005). In addition, the maternal immune system should also be modulated to tolerate the semi-allogeneic embryo (Finn, 1975; Trowsdale and Betz, 2006). However, the mechanisms responsible for maternal tolerance remain incompletely elucidated. Chemokines are a superfamily of small-molecule cytokines, widely expressed in trophoblast cells, decidual stromal cells (DSCs), and decidual immune cells (DICs) at the maternal–fetal interface. Emerging evidence has identified chemokines and chemokine receptors as essential contributors in pregnancy, participating in trophoblast invasion, decidualization, and immune cell recruitment (Hannan and Salamonsen, 2007; Ramhorst et al., 2016; Ashley et al., 2021). Moreover, the aforementioned functional abnormalities of chemokines have been reported in several pregnancy complications, including preeclampsia (PE), recurrent spontaneous abortion (RSA), and preterm birth (PTB) (Whitcomb et al., 2007; Kwak et al., 2014; Ali et al., 2021; Wang et al., 2021; Zheng et al., 2021). In this review, we summarized the crucial regulatory roles of chemokines in pregnancy in detail and highlighted their importance on specific cellular processes at the maternal–fetal interface. We also investigated the main chemokines and chemokine receptors related to pregnancy complications, hoping to provide a better understanding of these diseases.

## 2 Overview of Chemokines and Chemokine Receptors

Chemokines are a group of small secretory proteins of 8–10 kDa, well known for their chemotactic abilities (Zlotnik et al., 2006). Structurally, they are divided into the C chemokine ligand (XCL1-2), the CC chemokine ligand (CCL1-28), the CXC chemokine ligand (CXCL1-17), and the CX3C chemokine ligand (CX3CL). More than 50 chemokines have been identified since the late 1980s (Rollins, 1997; Zlotnik and Yoshie, 2000; Yoshie et al., 2001). They are widely expressed in humans (Sarkar et al., 2012), pigs (Hu et al., 2016), murine (Sarkar et al., 2012), and sheep (Ashley et al., 2011). Certain viruses also express molecules similar to chemokines (Penfold et al., 1999; Pontejo and Murphy, 2017; Pontejo et al., 2018). Correspondingly, there are about 20 chemokine receptors, including the CC chemokine receptor (CCR), the CXC chemokine receptor (CXCR), the C chemokine receptor (XCR), and the CX3CR chemokine receptor (CX3CR) (Zlotnik et al., 2006). In addition, there is a limited set of atypical chemokine receptors (ACKR1-4), which act as chemokine scavengers without eliciting chemotaxis (Stone et al., 2017). Together, chemokines and chemokine receptors constitute a rich chemokine system. It is generally accepted that there is a redundancy characteristic in the chemokine system. In other words, most chemokine receptors tend to bind to more than one ligand, and at the same time, a single ligand can also interact with different receptors. However, a recent study questions this general and oversimplified point of view (Ellwanger et al., 2020). Functionally, chemokines have been widely reported to be involved in inflammation, tumor, or metabolic diseases (Charo and Ransohoff, 2006; Nagarsheth et al., 2017; Chen et al., 2018).

## 3 Regulation and Function of Chemokines at the Maternal–Fetal Interface

**FIGURE 1:**
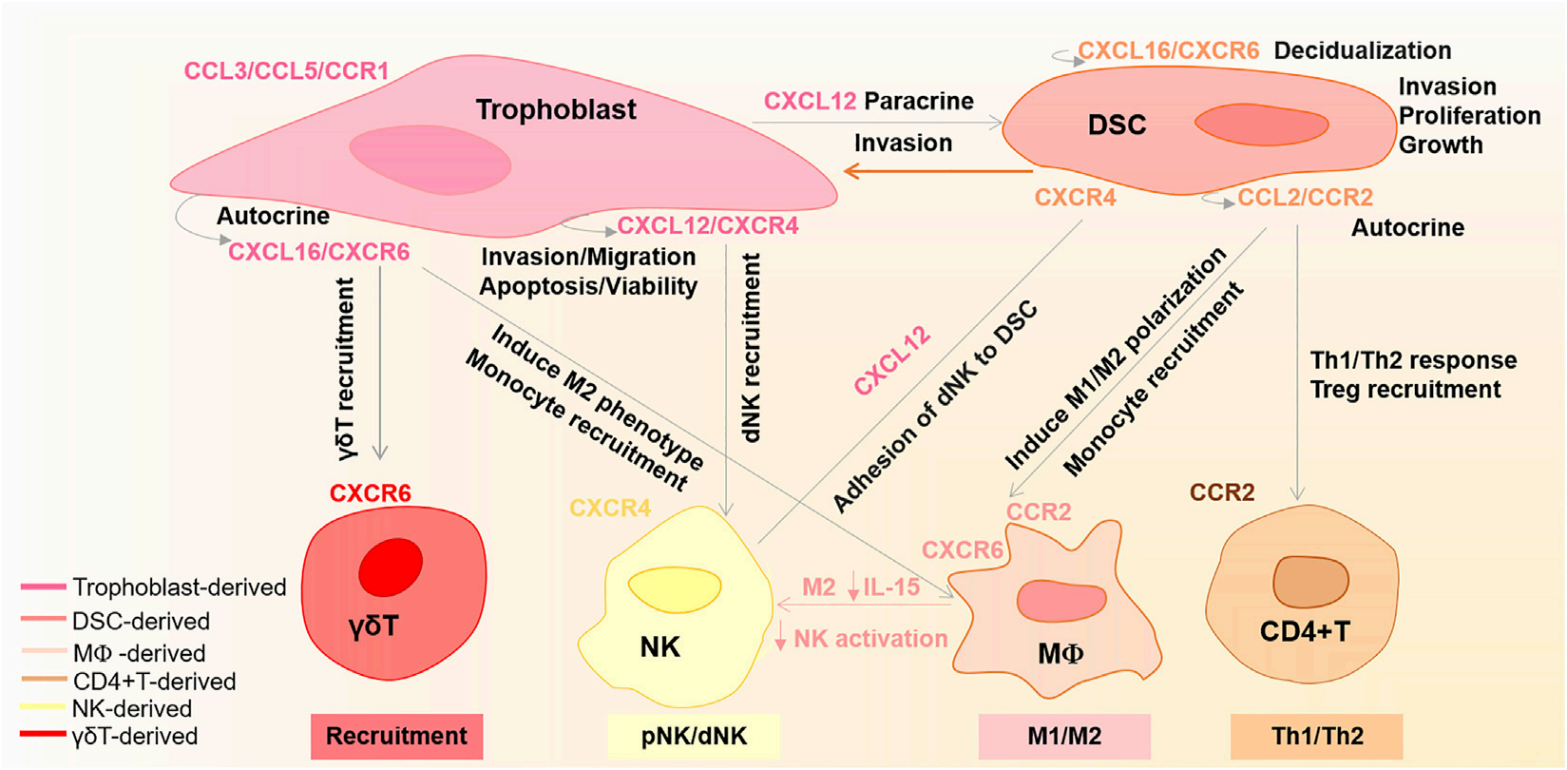
Regulation of main chemokines and chemokine receptors at the maternal–fetal interface. CXCL16 binding to CXCR6 promotes trophoblast invasion and proliferation, as well as endometrium decidualization in an autocrine and paracrine manner, respectively. CXCL16 is also involved in recruiting γδT cells and monocytes. Moreover, CXCL16/CXCR6 can induce M2 phenotype macrophages and decrease their IL-15 levels, which in turn induce the inactivation of NK cells. Trophoblast-derived CXCL12 with CXCR4 not only promotes trophoblast cell migration, invasion, and apoptosis *via* an autocrine manner but also enhances DSC invasion by upregulating the CXCR4 expression in a paracrine manner. Additionally, the trophoblast-derived CXCL12 also participates in NK cell recruitment and enhances the adhesive abilities of dNK cells to DSCs. CCL2/CCR2 enhances DSC invasion, proliferation, and growth in an autocrine manner. Moreover, CCL2/CCR2 also shows roles in M1/M2 phenotype polarization, monocyte recruitment, Treg recruitment, and Th1/Th2 immune response. CCL3/CCL5/CCR1 plays an essential function in trophoblast invasion.

In recent years, many studies have highlighted the importance of chemokines in pregnancy. Compared to non-pregnant endometrium, the decidual tissue shows increased chemokine levels (Engert et al., 2007; Segerer et al., 2009). During labor, some inflammatory chemokines are also upregulated in the uterus myometrium (Huang et al., 2021). At the maternal–fetal interface, the trophoblast cells, DSCs, and DICs establish rich chemokines and chemokine receptor network (Du et al., 2014). This chemokine network not only regulates specific recruitment and activation of appropriate leucocytes but also coordinates precisely orchestrated invasion of trophoblast through the decidua and maternal vasculature (Jones et al., 2004; Red-Horse et al., 2004; Hannan and Salamonsen, 2007; Fraccaroli et al., 2009). In this section, we will provide a detailed overview of the regulation and function of crucial chemokines at the maternal–fetal interface, as shown in Figure 1.

### 3.1 CCL3/CCL5/CCR1

Trophoblasts, which form at the early stages of pregnancy, subsequently differentiate along the villous or extravillous trophoblast (EVT) pathway. In detail, at the tip of the anchoring villi, the trophoblasts proliferate and differentiate into EVTs while on the border layer of the floating villi, they differentiate into syncytiotrophoblasts. The former participates in endometrium decidualization and spiral artery remodeling, while the latter is responsible for nutrition transport, waste elimination, and placental endocrine functions. All of these aforementioned processes are related to the trophoblast biological function of invasion, migration, proliferation, and apoptosis. Chemokines and chemokine receptors have shown crucial regulatory roles in these aspects (Fujiwara et al., 2005a). It has been found that trophoblasts can acquire chemokine receptors CCR1 as they differentiate into invasive EVTs. Sato et al. (2003) collected the human placental tissue at 9–10 weeks of gestation for immunohistochemical detection. They found that the EVTs highly expressed CCR1 while the syncytiotrophoblasts and cytotrophoblasts hardly expressed CCR1 (Sato et al., 2003). They further demonstrated that CCR1 combined with its ligand CCL5 or CCL3 promoted the migration of the EVTs, which were isolated from the explant cultures *in vitro* (Sato et al., 2003). The primary EVTs can also trap CCL5 from maternal platelets, thereby enhancing their invasion abilities (Sato et al., 2005; Sato et al., 2010; Sato, 2020). Consistently, in the first trimester of pregnancy, CCL3 also showed a rapid increase during trophoblast differentiation toward EVTs (Drake et al., 2001). In contrast, the expression of CCR1 showed a significant decrease after EVTs migrated to the decidua. Interestingly, Fujiwara et al. (2005b) found that the EVTs also expressed dipeptidyl peptidase IV to metabolize CCL5, therefore inhibiting the excessive cell invasion. This regulation of trophoblast invasion and differentiation function by the chemokine-CCR1 system is considered a key molecular mechanism of maternal vascular remodeling during human early pregnancy (Sato et al., 2012).

### 3.2 CXCL12/CXCR4/CXCR7

CXCL12, also known as stromal cell-derived factor 1, is initially discovered in the bone marrow–derived stromal cells (Balabanian et al., 2005). According to reports, CXCL12 is widely expressed in cytotrophoblasts, syncytiotrophoblasts, and EVTs (Douglas et al., 2001; Red-Horse et al., 2001; Wu et al., 2004). Its receptors CXCR4 and CXCR7 are detected in DSCs (Zhou et al., 2008), trophoblast cells (Wu et al., 2004), and decidual natural killer (dNK) cells (Tao et al., 2015). Studies have shown that the CXCL12/CXCR4/CXCR7 axis is the critical signaling component of pregnancy through participating in multiple processes at the maternal–fetal interface.

#### 3.2.1 Regulation and Function in Trophoblast Cells and Decidual Stromal Cells

The CXCL12/CXCR4/CXCR7 axis shows multiple roles in trophoblast cells during pregnancy. Wu et al. (2004) first isolated human placental trophoblast cells at 5–10 weeks of gestation, and they found that CXCL12/CXCR4 increased the trophoblast viability in an autocrine manner *in vitro*. Tripathi et al. (2014) reported that CXCL12/CXCR7 can promote JAR cell survival *in vitro*. Lu et al. (2016) observed that with decreased CXCR4 and CXCL12 levels, term human placental trophoblasts isolated from PE patients showed a significant apoptosis tendency, suggesting their roles in trophoblast apoptosis. Specific knockdown of CXCR4 in mouse trophectoderm cells of blastocysts significantly decreased the implantation rate of embryos (Bao et al., 2016). Further analysis indicated that CXCR4 is required upstream of trophectoderm cell apoptosis and migration (Bao et al., 2016). CXCL12/CXCR4 also shows regulation in trophoblast invasion. Both CXCR4 and CXCR7 showed increased expression during the cytotrophoblast differentiation toward the invasive phenotype (Schanz et al., 2011a). *In vitro* experiments showed that CXCR4 favored JEG-3 cell migration and invasion (Zhang et al., 2018). Correspondingly, downregulated CXCL12 showed direct suppression in HTR-8 cell invasion (Tamaru et al., 2015). In these aforementioned methods, CXCL12/CXCR4/CXCR7 widely regulates the trophoblast cell biological function.

The migratory and invasive capacities of human endometrial stromal cells (ESCs) are increasingly recognized as important features in the reproductive function (Weimar et al., 2013). Decidualized ESCs even perform enhanced motility and invasive capacity (Gellersen et al., 2010). By using an embryo coculture model, Grewal et al. (2008) reported this motility of decidualized ESCs. Interestingly, CXCL12 shows a functional role in DSC invasion. Ren et al. (2012) found that primary trophoblast-derived CXCL12 promoted the invasion of human first-trimester DSCs in a paracrine manner (Ren et al., 2012). Further investigation showed that this effect was mediated *via* CXCR4 but not CXCR7 (Zheng et al., 2018). CXCR7 may play the role of decoy in trophoblast invasion. At the same time, the invasiveness activity of trophoblast cells in coculture with DSCs also increased significantly and could be inhibited by an anti-CXCR4 neutralizing antibody (Zhou et al., 2008). These studies altogether suggest that CXCL12/CXCR4/CXCR7 not only participates in regulating trophoblast cells and the DSC biological function but also constructs a cross-talk between trophoblast cells and DSCs during pregnancy.

#### 3.2.2 Regulation and Function in Decidual Immune Cells

##### 3.2.2.1 Natural Killer Cells

Up to 70% of DICs are NK cells (King et al., 1989; Verma et al., 2000). In contrast to CD56^dim^CD16^+^ peripheral NK (pNK) cells, dNK cells are mainly CD56^bright^CD16^−^ cells (Jabrane-Ferrat, 2019). DNK cells have poor cytotoxic activity and are believed to be critical in maintaining maternal–fetal tolerance and placental vascular remodeling. CXCR4 is essential for the composition of dNK cells. Hanna et al. (2003) found that CXCR4 was preferentially expressed on CD16^−^ dNK subsets. Wu et al. (2005) also revealed that CD56^bright^CD16^−^dNK cells highly transcribed CXCR4. Moreover, CXCL12/CXCR4 shows crucial roles in regulating NK cell recruitment and differentiation during pregnancy. According to a previous report, the CXCL12/CXCR4 axis promoted the recruitment of CD25^+^ NK cells and the accumulation of CD3^−^ CD56^bright^CD25^+^ dNK cells at the maternal–fetal interface (Tao et al., 2015). Subsequently, Piao et al. (2015) found that it is the first-trimester human trophoblast-derived CXCL12 that induced pNK cell recruitment and differentiation toward dNK cells. A recent study also reported that trophoblast-derived CXCL12 enhanced the adhesive abilities of CD56^bright^CD82^−^CD29^+^ NK cells to DSCs *via* the CXCL12/CD82/CD29 signaling pathway and thus contributed to CD56^bright^ NK cell enrichment in decidua (Lu et al., 2020a). In particular, decidual CXCR4^+^CD56^bright^ NK cells have been identified as a novel NK subset, which plays vital immune-modulatory roles in the Th1/Th2 response. It has long been established that dynamic deviations in Th1 and Th2 profiles are closely associated with pregnancy maintenance (Raghupathy, 2001). In the initial stages of pregnancy, there is a clear need for an active Th1 inflammatory response to achieve embryo implantation (Granot et al., 2012). Subsequently, a continuous prevalence of anti-inflammatory Th2 bias helps the mother to accommodate the semi-allogeneic embryo until a progressive shift toward Th1 predominance for labor (Challis et al., 2009). According to the report, CXCR4^+^CD56^bright^ dNK cells can promote the Th2 shift in an IL-4-dependent manner (Tao et al., 2021). Diminished CXCR4^+^ dNK cells and their impaired ability to induce Th2 differentiation were already found in RSA patients and mouse models (Tao et al., 2021). Moreover, the adoptive transfer of CXCR4^+^ dNK cells to NK-deficient mice showed their great therapeutic potential in recovering the Th2/Th1 bias and reducing embryo resorption rates (Tao et al., 2021). Collectively, these studies suggest a crucial role of CXCL12/CXCR4 in NK cell recruitment and the Th1/Th2 response, providing the foundation for understanding the regulation of NK cells in maternal–fetal immune tolerance.

##### 3.2.2.2 T Cells

A previous study investigated the role of CXCL12 in T-cell recruitment and differentiation. According to this report, the percentage of embryo loss was markedly decreased in the pregnant non-obese diabetic mice by exogenous regulatory T (Treg) cell transfer along with a CXCL12 injection (Lin et al., 2009). Subsequent *in vitro* cell migratory experiments showed that T-cell migration cannot be detected when no CXCL12 was added beforehand. In contrast, a considerable percentage of T cells were attracted after CXCL12 addition (Lin et al., 2009). These results indicate that CXCL12 may regulate the migration of T cells into the pregnant uterus and differentiation toward Treg, therefore establishing a beneficial environment for allogeneic pregnant nonobese diabetic mice. The CXCL12/CXCR4 axis is also involved in the Th1/Th2 balance at the maternal–fetal interface in early human pregnancy. By the bioplex assay, Piao et al. (2012) found that human recombinant CXCL12 alone increased Th2-type IL-4 and IL-10 production while decreasing the Th1-type TNF-α expression in primary DICs isolated from the first-trimester decidua. Further anti-CXCR4 antibody re-treatment eliminated the effect of CXCL12 on cytokine production in DICs, suggesting that the CXCL12/CXCR4 axis is involved in the development of the Th2 bias at the maternal–fetal interface (Piao et al., 2012).

##### 3.2.2.3 Dendritic Cells

Dendritic cells (DCs) are a heterogeneous population and have a dual immune regulatory role. They not only initiate primary immune response but also induce immunological tolerance (Banchereau and Steinman, 1998). The immune-suppressive phenotype and function of DCs are critical for pregnancy (Blois et al., 2004; Bizargity and Bonney, 2009). However, research on human decidual DCs is quite sparse since the difficulty of small cell proportion and no single specific marker for DCs. Limited research reported the function of CXCL12/CXCR4 in DCs. Human monocyte-derived DCs can express CXCR4, responsible for chemotaxis to CXCL12. A subsequent study showed that CXCL12/CXCR4 can enhance DC maturation and survival to initiate acquired immune response in non-pregnant mice (Kabashima et al., 2007). Remarkably, impaired homing of CXCR4^+^ DCs during early gestation provoked a disorganized decidual vasculature with impaired spiral artery remodeling later (Barrientos et al., 2013). Conversely, the adoptive transfer of CXCR4^+^ DCs rescued early pregnancy (Barrientos et al., 2013).

### 3.3 CXCL16/CXCR6

#### 3.3.1 Regulation and Function in Trophoblast Cells and Decidualization

Huang et al. (2006a) detected that CXCL16 and CXCR6 are widely expressed in syncytiotrophoblasts, EVTs, and cytotrophoblasts of placentas at 7–9 weeks of gestation by immunohistochemistry. Moreover, they found that CXCR6/CXCL16 stimulated the first-trimester human trophoblast proliferation and invasion in an autocrine manner (Huang et al., 2006a). Moreover, CXCL16 can also upregulate the expression of antiapoptotic markers in trophoblast cells, suggesting its potential for trophoblast apoptosis (Fan et al., 2019). It has reported the role of CXCL16/CXCR6 in decidualization. Compared to ESCs, the primary human DSCs secreted and expressed higher CXCL16 and CXCR6 (Mei et al., 2019). Meanwhile, the decidualized ESCs showed a significant decidual response after being treated with exogenous recombinant human CXCL16 or trophoblast-secreted CXLC16 *in vitro*. These results indicated that the CXCL16/CXCR6 axis contributed to the progression of ESC decidualization (Mei et al., 2019).

#### 3.3.2 Regulation and Function in Decidual Immune Cells

##### 3.3.2.1 Macrophages

Macrophages are heterogeneous and are generally divided into two categories: classically activated macrophages (M1) and alternatively activated macrophages (M2) (Mills et al., 2000). Decidual macrophages perform a mixed immune status of M1 and M2 phenotypes according to reports. Successful pregnancy depends on the spatial and temporal balance of M1 and M2 polarization (Brown et al., 2014). This regulation of macrophages is susceptible to changes in the maternal–fetal microenvironment. It has been found that CXCL16/CXCR6 can regulate macrophage polarization (Mantovani et al., 2004; Yao et al., 2019). Wang et al. revealed that first-trimester human trophoblast-derived CXCL16 induced the M2 phenotype of macrophages *in vitro*. Moreover, the polarized M2 macrophages can downregulate IL-15 levels, thereby facilitating the inactivation of NK cells and contributing to the immunotolerance at the maternal–fetal interface (Wang et al., 2019). In addition, CXCL16 also exhibits its action in monocytes. By using an enzyme-linked immunosorbent assay (ELISA), Huang et al. (2008) first detected CXCL16 secretion in the conditioned medium of primary cytotrophoblasts isolated from villi at 7–9 weeks of gestation (Huang et al., 2008). Next, by flow cytometry, they demonstrated that both exogenous and cytotrophoblast-conditioned medium-derived CXCL16 can direct the migration and recruitment of the monocyte subtype in peripheral blood mononuclear cell or decidua leukocytes (Huang et al., 2008). This chemotactic response of monocyte subtypes to CXCL16 was largely parallel to their receptor CXCR6 expression (Huang et al., 2008). These findings suggest that the fetus-derived trophoblasts can attract monocytes by CXCL16/CXCR6 in the first-trimester pregnancy, forming a specialized immune milieu at the maternofetal interface.

##### 3.3.2.2 T Cells

CXCL16/CXCR6 can recruit and migrate T cells toward decidua, participating in the immune regulation of pregnancy. Using multiple-color flow cytometry, Huang et al. (2008) demonstrated that the CXCL16 sole receptor CXCR6 is preferentially expressed on decidual γδT cells (Huang et al., 2008). Furthermore, they confirmed that fetal trophoblast-produced CXCL16 directed the migration and recruitment of peripheral and decidual T lymphocytes into decidua at 7–9 weeks of gestation, thereby leading to a specialized immune milieu formation at the maternal–fetal interface (Huang et al., 2008). Fan et al. (2019) reported that by reducing the secretion of the cytotoxic factor granzyme B of decidual γδ T cells, the CXCL16/CXCR6 axis may contribute to maintaining normal pregnancy.

### 3.4 CCL2/CCR2

Previous research has showed that the primary trophoblasts did not express CCR2, while the primary isolated human DSCs highly transcribed CCR2 (Wu et al., 2004). He et al. (2007) found the co-expressions of CCR2 and CCL2 in human first-trimester DSCs and the decidual tissue. They detected high levels of CCL2 secretion in the supernatant of primary DSCs with an ELISA (He et al., 2007). Subsequently, Meng et al. (2013) reported that elevated CCL2/CCR2 promoted primary human DSC proliferation and growth. Hu et al. (2014) found that upregulated CCL2/CCR2 enhanced primary human DSC invasion. These two studies together emphasized the function of CCL2/CCR2 on DSC invasion, proliferation, and growth. In addition, CCL2/CCR2 also shows key functions in DICs. Wei et al. (2021) found that CCL2/CCR2 determined the polarization phenotype of decidual macrophages in a monocyte-DSC coculture system in a paracrine manner during early pregnancy. They detected changes both in decidual macrophages’ percentage and the M1 and M2 marker expressions after treatment with the CCR2 inhibitor by the flow cytometry assay *in vivo* (Wei et al., 2021). Another report exhibited that the first trimester decidual cell-derived CCL2 promoted monocyte migration and thus mediated excessive macrophage infiltration of the decidua (Huang et al., 2006b). CCL2 also shows an indirect role in the T-cell response. Huang et al. (2020)reported that human chorionic gonadotropin promoted the recruitment of regulatory T cells in the endometrium through increasing CCL2 levels in human ESCs. Yu et al. (2021) revealed that Toll-like receptors induced Th1/Th2 responses by affecting the CCL2 secretion of DSCs at the maternal–fetal interface.

## 4 Chemokines and Pregnancy Complications

The abnormal expression of chemokines and chemokine receptors can interrupt the trophoblast function, uterus angiogenesis, and maternal–fetal immune tolerance, thereby participating in pregnancy complications (Hannan and Salamonsen, 2007). In this part, we will focus on the relationship between chemokines and pregnancy-associated diseases, including PE, RSA, and PTB.

### 4.1 Preeclampsia

PE is defined as hypertension after 20 weeks of gestation and proteinuria with maternal multisystem dysfunction or fetal growth restriction (Chappell et al., 2021). PE is a major cause of maternal and perinatal mortality and morbidity, affecting approximately 5% of pregnancies (Bibbins-Domingo et al., 2017). However, the pathological mechanism of PE remains unclear. A prevailing view holds that PE is related to inadequate trophoblast invasion and placental malperfusion with releasing of soluble factors into the circulation, which causes maternal vascular endothelial injury and further leads to hypertension and multi-organ dysfunction (Chappell et al., 2021). CXC chemokines have unique abilities in angiogenesis and trophoblast function and are believed to play a potential role in the pathogenesis of PE. Decreased placental CXCL3 damaged the invasion and angiogenesis of trophoblast, thus leading to shallow implantation, which may be the main cause of severe PE (Gui et al., 2014; Wang et al., 2018). Reports are conflicting about the role of CXC12/CXCR4/CXCR7 in PE. Previous research has reported higher CXCL12 levels in the placenta of PE patients compared to the normal control group (Schanz et al., 2011b; Hwang et al., 2012). However, recent research has showed that CXCL12 and its receptors CXCR4 and CXCR7 levels were downregulated in the placenta of severe PE patients (Lu et al., 2016). Further studies have found that CXCL12 was able to decrease term trophoblast cells’ apoptosis rate (Lu et al., 2016; Lu et al., 2020b). Therefore, downregulation of CXC12/CXCR4/CXCR7 may disturb trophoblast apoptosis, participating in the occurrence of severe PE. Remarkably, CXCL12 levels were elevated in the mid-trimester amniotic fluid of pregnant women with PE, while the mechanism remains unknown (Tseng et al., 2009). In summary, these findings suggest that the CXCL12/CXCR7/CXCR4 axis may be a crucial molecular clue of PE that is worth to be further studied. Interestingly, the chemokines also show their effects on DICs in PE (Valencia ‐ Ortega et al., 2020). Excess CXCL10 and CXCL11 in decidua blunted pNK cell recruitment, contributing to the genesis of shallow placentation in PE (Lockwood et al., 2013). The increased CCL2 in first-trimester decidual cells showed association with the accumulation of decidual macrophages in the preeclamptic decidua (Lockwood et al., 2006).

### 4.2 Recurrent Spontaneous Abortion

RSA is defined as two or more times consecutive miscarriages before 20 weeks of gestation and impacts approximately 5% of childbearing-age women (Pereza et al., 2017; Practice Committee of the American Society for Reproductive Medicine, 2020). A recent transcriptomic analysis proposed chemokines as a common pathogenic mechanism in pregnancy loss (Wang et al., 2021). Abnormal secretion of CXCL5 was reported as an early indicator of miscarriage risk (Whitcomb et al., 2007). Recent research also showed that CXCL5 levels were downregulated in villous tissue of RSA patients than those of the controls (Zhang et al., 2021). Upregulation of CXCL5 can lead to poor trophoblast invasion and thus may be correlated with RSA (Zhang et al., 2021). CCR7 levels showed a decrease in the villous of RSA women (Luan et al., 2020). Knockdown of CCR7 caused an obvious reduction of migration and invasion in JAR and JEG-3 cells (Luan et al., 2020). These studies suggest that the chemokine system-induced trophoblast invasion dysfunction may be a potential pathological mechanism of RSA. CXCL12/CXCR4 also showed important roles in pregnancy loss. Das et al. found significantly reduced CXCR4 levels in chorionic villi of women with a number of previous miscarriages (Zangmo et al., 2021). This may induce insufficient trophoblast invasion, defective decidualization, or an imbalance of maternal–fetal immune tolerance and thus act on miscarriages (Ren et al., 2012; Piao et al., 2015; Ao et al., 2020). Women who exhibited recurrent implantation failure also performed lower levels of CXCR4 in the endometrium compared with fertile women (Tapia et al., 2008). Interestingly, CXCL12 from bone marrow-derived cells or the stem cells can improve the thin endometrium in a mouse model (Yi et al., 2019). Intrauterine CXCL12 administration in C57BL/6 mice also promoted embryo implantation rates and induced endometrial angiogenesis *in vitro* (Koo et al., 2021). These studies suggested that CXC12/CXCR4 may act on the endometria and angiogenesis, mediating its role in pregnancy loss (Wang et al., 2015). Some studies have also reported decreased CXCL16 protein levels in the villus of RSA patients compared with normal pregnant women (Fan et al., 2019; Mei et al., 2019). However, the fact whether the abnormal expression of CXCL16 at the maternal–fetal interface is the cause of miscarriage remains unclear. Kuroda et al. (2021) reported the relationship between the increasing number of pregnancy losses and the elevated ratio of Th1/Th2 in blood samples. Moreover, the ratio of Th1 and Th2-related chemokine receptors seems to have a crucial association with RSA. By flow cytometry, Kheshtchin et al. (2010) analyzed the expression of Th1-related (CCR5 and CXCR3) and Th2-related (CCR3 and CCR4) chemokine receptors on peripheral CD4^+^ or CD8^+^ T cells from RSA and control group women before 20 weeks of gestation. They reported a higher ratio of Th1/Th2 chemokine receptors in RSA women, indicating the Th1 dominant immune responses in the circulation of RSA women (Kheshtchin et al., 2010). Compared with fertile women, chemokine CCL5 performed decreased serum levels in patients with RSA while increased after immunization with paternal leukocytes. CCL5 can inhibit the mixed lymphocyte reaction in a dose-dependent manner *in vitro* (Ramhorst et al., 2004). These studies emphasized that the chemokines may exert immunological effects and thus take part in RSA. However, these aforementioned studies are only based on peripheral blood data, and further studies on the maternal–fetal interface are necessary.

### 4.3 Preterm Birth

PTB is defined as giving birth to babies before 37 weeks of gestation and is the leading cause of perinatal morbidity and mortality in developed countries (Goldenberg et al., 2008). Multiple factors attribute to the occurrence of PTB, such as inflammation, stress, and hormonal disorders (Romero et al., 2006; Goldenberg et al., 2008). Aberrant levels of chemokines have been reported in women with PTB. By ELISA, Laudanski et al. (2014) detected unusually high CCL16 levels in the blood serum samples of women with PTB. Subsequently, another study showed that lower CCL16 in umbilical cord blood was associated with spontaneous PTB, with 94.7% prediction sensitivity and 46.9% specificity (Kaukola et al., 2011). This indicates that CCL16 may be one of the potential pathological factors of PTB. Studies of chemokines in the amniotic fluid of women with PTB provide additional clues. For example, increased CXCL10 in the amniotic fluid showed the risk of spontaneous PTB after 32 weeks of gestation (Gervasi et al., 2012). Other studies also reported that CXCL12 (Tseng et al., 2009), CXCL8 (Hamilton et al., 2013), CCL5 (Hamilton et al., 2013), CCL20 (Hamill et al., 2008; Hua et al., 2012), CXCL5 (Hua et al., 2012), and CCL7 (Jacobsson et al., 2005) in the amniotic fluid were associated with microbial invasion and amniotic cavity inflammation. Studies from animals showed that broad-spectrum chemokine inhibitors can inhibit infection-mediated PTB (Shynlova et al., 2014; Coleman et al., 2020). These reports suggest that the chemokines may be involved in PTB through the inflammatory response. However, Esplin et al. (2005) found that CCL2 was increased in the amniotic fluid of PTB women with or without intra-amniotic infection. Interestingly, a prospective immunohistochemical analysis of 203 chorionic villus sampling specimens showed that the scores of syndecan-1, a regulator of chemokine function, are correlated with PTB (Schmedt et al., 2012). Recently, a transcriptomic analysis has also reported that the chemokine pathway may present a common pathogenic mechanism in spontaneous PTB (Wang et al., 2021). Taken together, these studies implied that chemokines play important roles in the pathological mechanism of PTB.

## 5 Conclusion

Successful pregnancy requires participation and cooperation of multiple crucial events, including good trophoblast function, decidualization, and balanced maternal–fetal immune tolerance. In this review, the available evidence shows that chemokines and chemokine receptors have wide regulatory effects in these events surrounding the trophoblast cells, DSCs, and DICs. Abnormalities of chemokines are related to trophoblast dysfunction, impaired angiogenesis, and disturbances in the maternal–fetal immune tolerance, which therefore may lead to pregnancy complications. Reviewing the regulation and function of chemokines in pregnancy may provide some potential targets for the clinical treatment of abnormal pregnancies in the future.
